# Necrotic pancreatitis: modern approaches to the prevention of infected necrosis

**DOI:** 10.3389/fmed.2025.1695035

**Published:** 2026-01-13

**Authors:** Murat K. Jakanov, Gulnur G. Gainollina, Bazylbek S. Zhakiev, Mikhail A. Topchiev, Bulat A. Kurmanbayev

**Affiliations:** 1 Department of General Surgery, West Kazakhstan Marat Ospanov Medical University, Aktobe, Kazakhstan; 2 Department of Surgical Diseases No. 2, West Kazakhstan Marat Ospanov Medical University, Aktobe, Kazakhstan; 3 Department of General Surgery with Postgraduate Education, Astrakhan State Medical University of the Ministry of Health, Astrakhan, Russia

**Keywords:** acute pancreatitis, necrotizing pancreatitis, infected necrosis, antibiotic prophylaxis, infection prevention

## Abstract

The investigation of etiological factors in acute pancreatitis is a critical task, as their elucidation contributes to advancing both the diagnosis and prevention of the disease. Necrotizing pancreatitis (NP) is a severe complication of acute pancreatitis associated with high morbidity and mortality due to enzymatic tissue destruction, systemic inflammation, and the risk of infected necrosis. This review synthesizes current knowledge on the pathogenesis, classification, and epidemiology of NP, with emphasis on strategies aimed at preventing infectious complications. Evidence consistently supports the protective role of early enteral nutrition, while the use of selective digestive decontamination and antibiotic prophylaxis remains debated. Although several clinical trials and meta-analyses have explored antibiotic prophylaxis, results are inconclusive, reflecting heterogeneity in study design, antibiotic regimens, and patient selection. Recent advances in risk stratification, including the application of machine learning models, offer promising tools for identifying patients at highest risk for infection and tailoring preventive interventions. Nevertheless, important knowledge gaps remain, particularly regarding the optimal timing, duration, and selection of prophylactic measures. Current data suggest that prevention strategies should integrate nutritional support, judicious antimicrobial use, and individualized risk prediction. In conclusion, while significant progress has been made in understanding NP and developing preventive approaches, the efficacy of antibiotic prophylaxis continues to be controversial. Future large-scale, methodologically rigorous studies are needed to establish standardized preventive protocols and ultimately improve clinical outcomes in patients with necrotizing pancreatitis.

## 1 Definition and etiology of acute pancreatitis

The pancreas is a vital organ combining exocrine and endocrine functions, including secretion of digestive enzymes and hormones. Acute pancreatitis (AP), the most common pancreatic disorder, is caused by premature activation of digestive enzymes leading to autodigestion and inflammation. The investigation of etiological factors is critical for diagnosis, prevention, and individualized management.

**Figure 1:**
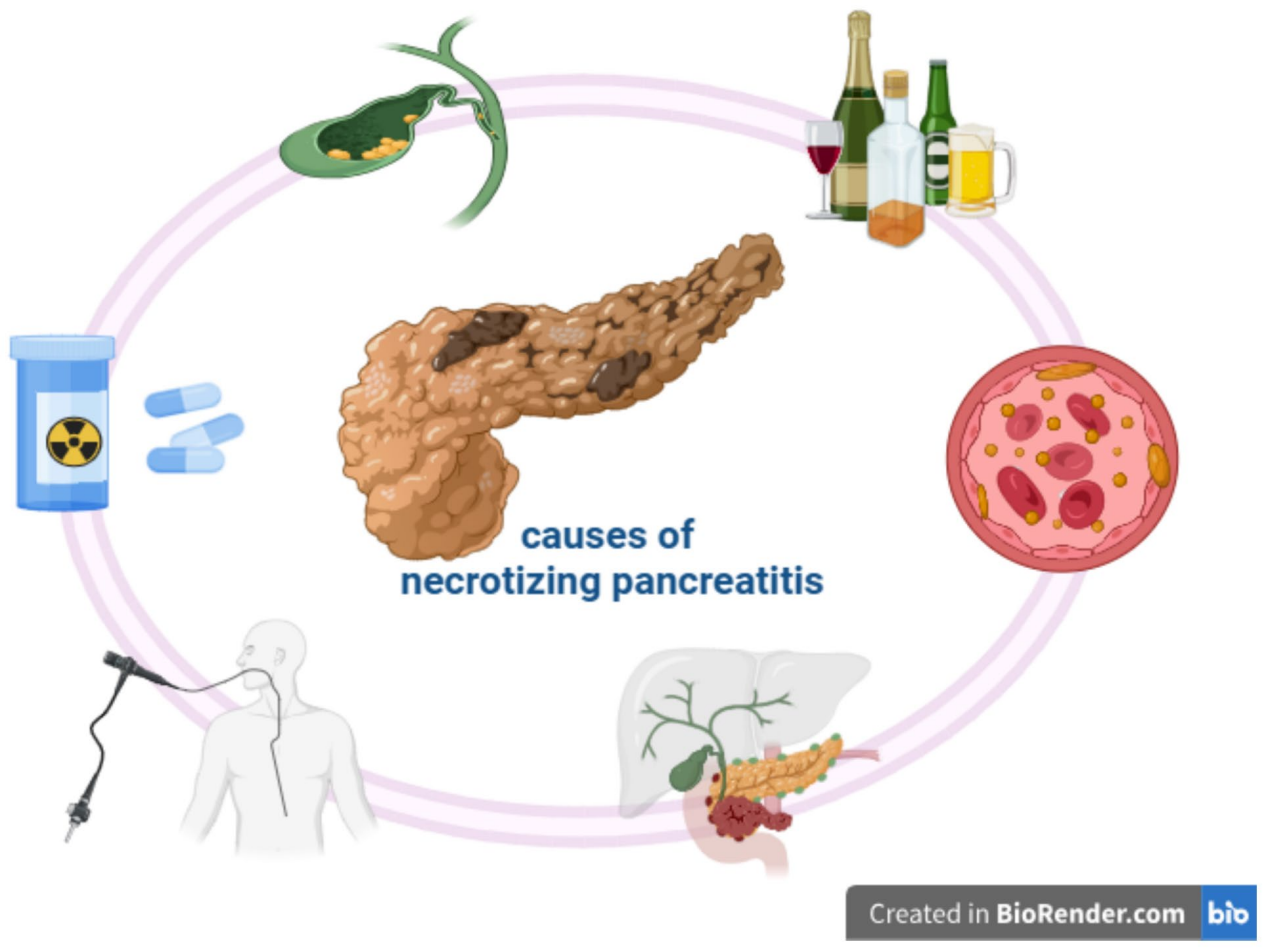
Causes of pancreatic necrosis.

Gallstone disease and alcohol consumption remain the predominant causes worldwide, accounting for 38–70% and 25–41% of cases, respectively. Hypertriglyceridemia represents approximately 10% of cases, but its prevalence is increasing in parallel with metabolic syndrome and obesity, underscoring the need for greater clinical awareness (1–3). Less common causes include pancreatic and periampullary tumors, abdominal trauma, and adverse drug reactions, such as azathioprine, furosemide, and corticosteroids (4). Iatrogenic pancreatitis, particularly after endoscopic retrograde cholangiopancreatography, also represents a relevant clinical problem (Figure 1) (5).

Although these etiological patterns are well established, their relative contribution differs across regions, reflecting variations in lifestyle, health system practices, and diagnostic capacity. Furthermore, in up to 20% of cases the etiology remains idiopathic despite advanced imaging and laboratory testing, highlighting persistent gaps in current diagnostic strategies (4).

## 2 Pathogenesis

Necrotizing pancreatitis arises from the premature and uncontrolled activation of digestive enzymes within pancreatic acinar cells (6). Under physiological conditions, trypsinogen is converted to trypsin only in the duodenal lumen; however, in pathological states, its intrapancreatic activation initiates a cascade involving other proteolytic and lipolytic enzymes, such as elastase and phospholipase A2, as well as the kallikrein–kinin system. This enzymatic activation results in direct cytotoxic damage to the pancreatic parenchyma and triggers the release of pro-inflammatory mediators, including interleukins (IL-1, IL-6, IL-8), tumor necrosis factor-*α* (TNF-α), and reactive oxygen species (7, 8). Collectively, these mediators amplify the local inflammatory response, promote tissue edema, and drive progressive necrosis.

The inflammatory cascade extends beyond the pancreas, causing systemic microcirculatory disturbances that critically shape disease severity. Cytokine-mediated increases in vascular permeability promote massive fluid extravasation into the retroperitoneal space and peritoneal cavity. Concurrently, endothelial activation, enhanced leukocyte adhesion, and impaired microvascular perfusion precipitate ischemia and accelerate pancreatic tissue necrosis. The spillover of cytokines and proteolytic enzymes into the systemic circulation induces systemic inflammatory response syndrome (SIRS), manifested by vasoplegia, hypovolemia, arterial hypotension, and a markedly increased risk of acute respiratory distress syndrome (ARDS) and multiple organ failure (MOF) (9, 10). Hemodynamic and inflammatory alterations are compounded by fluid sequestration within the intestinal lumen, enzymatic ascites, and intra-abdominal collections, which elevate intra-abdominal pressure (11). This contributes to impaired perfusion of vital organs (liver, kidneys, intestines), reduced venous return, decreased cardiac output, and respiratory compromise due to diaphragmatic elevation (12).

In later stages, sterile necrosis may be secondarily infected by translocation of enteric bacteria, leading to infected pancreatic necrosis, abscess formation, and sepsis. Such infectious complications substantially worsen prognosis and remain a leading cause of mortality (13). In summary, the pathogenesis of necrotizing pancreatitis is a multifactorial process encompassing premature enzymatic activation, inflammatory mediator release, microcirculatory dysfunction, systemic inflammatory response, and the potential superinfection of necrotic tissue.

## 3 Classification

**Figure 2:**
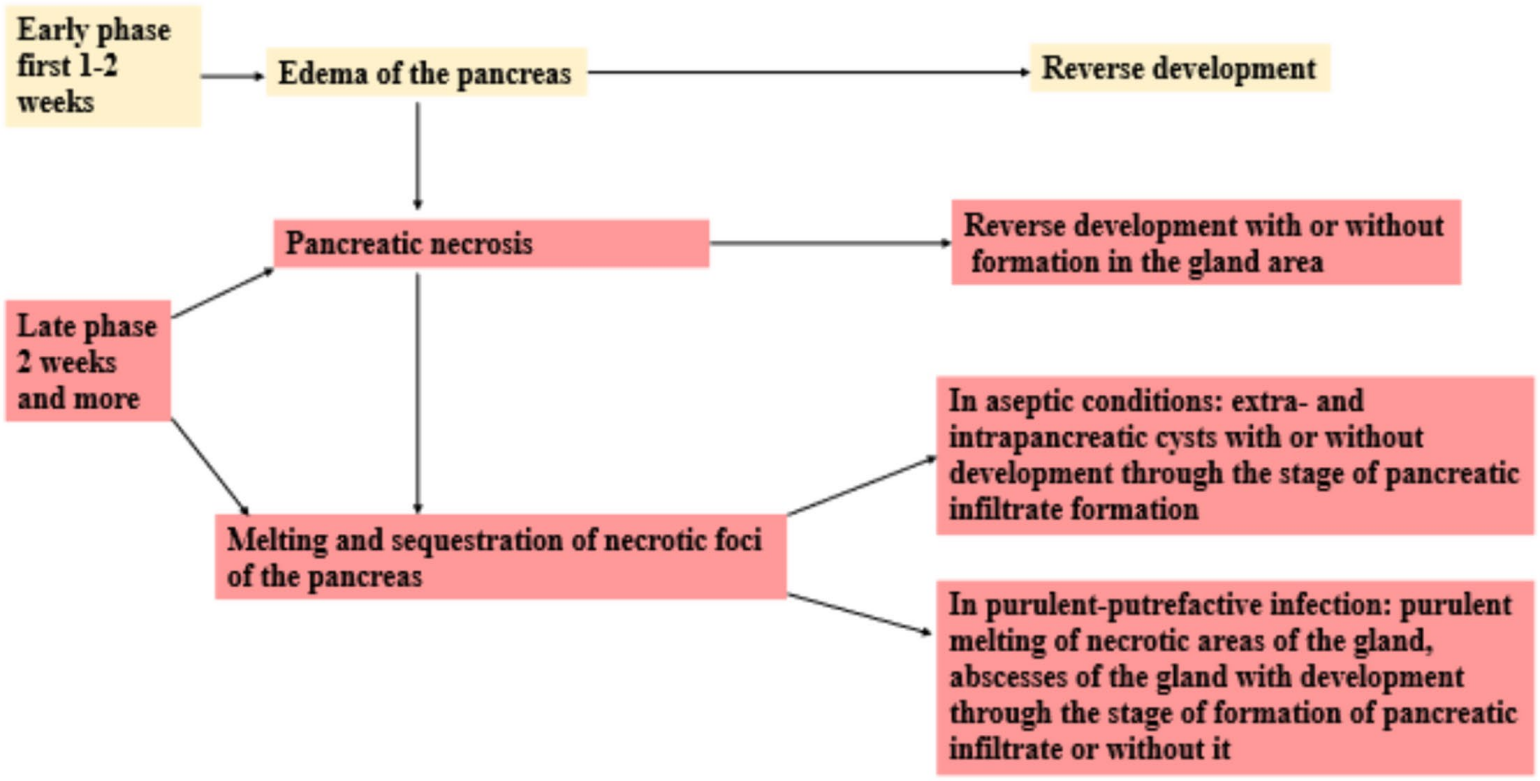
Phase development of acute pancreatitis.

The classification of AP was first proposed in 1992 and later revised in 2012 in what is now known as the Revised Atlanta Classification, developed through international consensus of specialists in pancreatic disease. AP typically evolves in two phases: an early phase (first 1–2 weeks) and a late phase (starting thereafter). While the early phase often lasts up to 1 week, the late phase may extend for weeks to months and is typically seen in patients with moderately severe or severe disease (Figure 2) (14, 15).

Based on imaging findings, local complications are categorized as interstitial edematous pancreatitis (IEP) or necrotizing pancreatitis. Four types of fluid collections are distinguished: acute peripancreatic fluid collections (APFCs) and pseudocysts, characteristic of IEP and containing only fluid and acute necrotic collections (ANCs) and walled-off necrosis (WON), which occur exclusively in necrotizing pancreatitis and include both fluid and necrotic debris (15). APFCs and pseudocysts are characteristic of interstitial edematous pancreatitis (IEP) and contain only fluid. In contrast, ANCs and WON occur exclusively in necrotizing pancreatitis and consist of both fluid and necrotic material (16). Necrotizing pancreatitis is accompanied by inflammation that leads to necrosis of the pancreatic parenchyma and/or peripancreatic tissue (15). Necrotizing pancreatitis may involve necrosis of pancreatic parenchyma and/or peripancreatic tissues.

In terms of severity, AP is divided into mild, moderately severe, and severe forms. Mild AP is characterized by absence of organ dysfunction and local or systemic complications, often resolving within a week (15, 17). Moderately severe AP involves transient organ dysfunction (resolving within 48 h) and/or local or systemic complications without persistent organ failure. Severe AP is defined by persistent organ failure lasting longer than 48 h and may involve either sterile or infected necrosis (15, 18).

Although the Revised Atlanta Classification remains the standard in both research and clinical practice, recent reviews have proposed augmentations toward an “Atlanta 2.0” that integrate advanced imaging-based staging and biomarker-informed stratification to capture heterogeneity and improve outcome prediction (19). Such proposals reflect the evolving understanding of AP and underscore the need for dynamic classification systems that better align with modern diagnostic and prognostic tools.

## 4 Epidemiology

Approximately 80% of AP cases are mild and self-limiting, with patients recovering without major complications. However, around 20% of patients experience a more complicated and prolonged course, often characterized by pancreatic necrosis. Of those with necrotizing pancreatitis, approximately 20–30% develop infected pancreatic necrosis (IPN), a serious and potentially life-threatening complication (20, 21).

Over the past decades, the global incidence of acute pancreatitis has shown a consistent upward trend. A meta-analysis of population-based studies reported an average annual percent change (AAPC) of 3.07% (95% CI 2.30–3.84) from 1961 to 2016, with the sharpest increases observed in North America and Europe, while data from Asia, Latin America, and Africa remain scarce and heterogeneous (22). The global burden study (23) further confirms rising regional incidence with projections indicating continued increase through 2050.

Notably, regional differences are striking: many epidemiological studies originate from high-income regions, while data from Asia, Latin America, Africa, and Central Asia remain limited. Complicated acute pancreatitis, particularly in cases of infected necrosis or persistent organ failure, is associated with mortality rates in the approximate range of 20–40%, depending on patient population, healthcare resources, and geography (24). In high-income settings such as North America and Europe, mortality is generally reported between 18 and 20%. By contrast, in China, reported rates range more widely, from 8 to 39%, while in Japan they are estimated at 10 to 26% (25).

## 5 Clinic and diagnostics

According to the Revised Atlanta Classification, the diagnosis of acute pancreatitis (AP) is established when at least two of the following criteria are present: (1) typical upper abdominal pain, (2) serum amylase or lipase levels more than three times the upper limit of normal, and (3) characteristic imaging features of AP on contrast-enhanced computed tomography (CECT), magnetic resonance imaging (MRI), or ultrasonography (15).

A detailed medical history and physical examination are crucial for identifying the underlying etiology, as management may differ depending on the cause. Important aspects of the anamnesis include previous episodes of pancreatitis and their complications, history of gallstone disease, prior biliary or pancreatic surgery, medication use (particularly drugs associated with pancreatitis such as azathioprine, furosemide, and corticosteroids), alcohol consumption, recent abdominal trauma, unexplained weight loss, and a family history of pancreatic disorders (26).

Laboratory testing should be performed within the first 24 h of admission and include amylase and lipase, liver function tests (to detect biliary obstruction), calcium, and triglycerides (27). While amylase is less specific, lipase is considered more sensitive and remains elevated longer, making it the preferred diagnostic biomarker (28). In addition, inflammatory biomarkers such as C-reactive protein (CRP > 150 mg/L at 48 h) and procalcitonin provide prognostic information and may predict the development of severe or infected pancreatitis (29).

Imaging plays a central role in diagnosis and assessing complications. Ultrasonography is recommended as the first-line modality in the initial work-up to detect gallstones, biliary obstruction, or peripancreatic fluid collections (27). CECT is considered the gold standard for identifying necrosis, characterizing fluid collections, and assessing disease severity; however, it should not be performed earlier than 72 h after symptom onset to avoid underestimation of necrotic changes (30). MRI and magnetic resonance cholangiopancreatography (MRCP) are useful alternatives for characterizing fluid collections and evaluating ductal anatomy, especially in patients with contraindications to contrast-enhanced CT (31). Endoscopic ultrasound (EUS) has high sensitivity for microlithiasis, small biliary stones, and pancreatic tumors and is recommended in patients with suspected biliary etiology when other modalities are inconclusive (32).

## 6 Prognosis and prevention

Accurate prediction of the clinical course and outcomes of AP remains a critical component of patient management. Over the past decades, several multifactorial scoring systems based on clinical, laboratory, and imaging data have been developed to stratify disease severity and guide therapeutic decisions. Among the most widely used are the Acute Physiology and Chronic Health Evaluation II (APACHE II) the Bedside Index for Severity in Acute Pancreatitis (BISAP) [46], the Modified Glasgow Score and Ranson’s criteria, as well as systemic inflammatory markers such as the Systemic Inflammatory Response Syndrome (SIRS) score (33, 34). Imaging-based indices, particularly the Modified Computed Tomography Severity Index (CTSI), provide valuable information on local complications and extent of pancreatic necrosis (35). APACHE II and BISAP are considered the most reliable scoring systems for predicting the severity of acute pancreatitis, although some experts emphasize that physician judgment and simple assessment of the Systemic Inflammatory Response Syndrome (SIRS) may be equally effective (35–37). SIRS is diagnosed when at least two of the following are present: temperature <36 °C or >38 °C, heart rate >90 beats per minute, respiratory rate >20 breaths per minute (or PaCO₂ < 32 mmHg), and white blood cell count <4,000 or >12,000/mm^3^ (34).

In recent years, machine learning (ML) has been increasingly applied to improve prognostic accuracy in AP (38, 39). ML models are capable of integrating large amounts of heterogeneous clinical and biochemical data, and systematic reviews have demonstrated their potential superiority over traditional scoring systems. A recent systematic review and meta-analysis (33 studies) demonstrated that machine learning approaches achieve a pooled C-index of ~0.87–0.88 in both training and validation sets for predicting severity in AP, however, predictive performance varied considerably across model types (40).

Alongside prognostic scores and computational models, several laboratory and immunological biomarkers are under investigation. Traditional markers such as C-reactive protein, blood urea nitrogen, urea concentration, and procalcitonin are frequently employed for early risk assessment (41, 42). Interleukins (IL-6 and IL-8) have been associated with the development of infected pancreatic necrosis (IPN), while biomarkers of immune suppression, such as interferon-*γ* and HLA-DR expression on monocytes, have been proposed as indicators of host response (9, 43). Recent data from the multicenter TRACE trial suggest that absolute lymphocyte count (ALC) may also serve as a useful predictor of pancreatic necrosis infection. However, many of these biomarkers are not yet widely available in clinical practice, limiting their utility outside of research settings (44).

Despite the significant progress in prognostic research, there is still debate regarding its clinical value. In a recent editorial, Edward L. Bradley III (45) called for an end to the proliferation of prognostic indices, arguing that research should instead focus on the etiology and pathogenesis of severe AP in order to develop more targeted treatment strategies. This reflects a growing recognition that, while accurate prediction is important, it should ultimately serve as a tool for improving therapeutic decision-making and patient outcomes rather than an end in itself.

### 6.1 Early nutrition

Current guidelines from the American Gastroenterological Association (46) recommend early oral or enteral feeding in acute pancreatitis (AP), emphasizing the preservation of gut integrity and reduction of bacterial translocation. While this approach contrasts with the traditional “bowel rest” strategy, clinical evidence increasingly supports its superiority. A meta-analysis by Ping Wu et al. (47) evaluated 11 RCTs with 562 patients and demonstrated that enteral nutrition (EN) significantly reduced mortality (RR = 0.43) and infectious complications (RR = 0.53) compared with parenteral nutrition (PN). However, the effect on multiple organ failure was not statistically significant (RR = 0.63), suggesting that EN primarily influences infection-related outcomes rather than systemic organ dysfunction (47). Additionally, EN shortened hospital stay, highlighting its potential to reduce healthcare burden.

Taken together, these findings support guideline recommendations and provide a mechanistic rationale: early EN maintains mucosal barrier function and mitigates bacterial translocation, which may reduce the progression to infected necrosis. Nevertheless, heterogeneity among studies—such as differences in patient severity, timing of intervention, and nutrition protocols—warrants cautious interpretation and indicates a need for further large-scale trials to refine optimal feeding strategies (48).

### 6.2 Probiotics

Probiotics, defined as non-pathogenic microorganisms that modulate the gut microbiome, were initially investigated as a potential therapeutic strategy in acute pancreatitis due to their presumed benefits in restoring intestinal barrier function, reducing pathogenic bacterial load, and promoting immunomodulation. Early studies suggested potential clinical benefits, but these findings were limited in scale and remain controversial (49).

However, the large multicenter, double-blind, placebo-controlled PROPACT trial Besselink et al. (50) involving 296 patients provided strikingly different results. In this study, probiotics were associated with a higher mortality rate (16% vs. 6% in the placebo group; RR 2.53, 95% CI 1.22–5.25), primarily due to intestinal ischemia, and did not reduce infectious complications (50). Follow-up investigations by Besselink et al. (51) confirmed these findings, demonstrating worsened enterocyte function and increased bacterial translocation in patients with organ failure.

Taken together, these results underscore that, despite a sound mechanistic rationale, probiotics can be harmful in severe acute pancreatitis. Current evidence therefore does not support their routine use, highlighting the need for caution in translating experimental therapies into clinical practice. This sequence of findings illustrates the importance of large, well-designed trials to validate preliminary positive results from smaller studies.

### 6.3 Preventive antibiotic therapy

In necrotizing pancreatitis, infection of pancreatic necrosis is a major determinant of morbidity and mortality. Since bacterial translocation from the intestines is considered a primary source of infection, prophylactic strategies have focused on limiting bacterial overgrowth, preserving gut barrier function, and preventing systemic infection (52, 53). Early randomized studies and systematic reviews initially suggested a potential benefit of antibiotic prophylaxis, particularly beta-lactams, in reducing mortality and the incidence of infected pancreatic necrosis (IPN) (54, 55). However, the reliability of these early findings was limited by small sample sizes and heterogeneous study designs.

Subsequent larger meta-analyses produced conflicting results. Ukai et al. (56), including six RCTs with 397 patients, reported that early antibiotic prophylaxis reduced both mortality (7.4% vs. 14.4%; OR 0.48, 95% CI 0.25–0.94) and the incidence of IPN (16.3% vs. 25.1%; OR 0.55, 95% CI 0.33–0.92), suggesting potential benefit when administered early. In contrast, Ding et al. (57), analyzing 11 RCTs with 747 patients, found no significant differences in mortality, IPN incidence, or need for surgical intervention, although extrapancreatic infections were slightly reduced in the prophylaxis group. Further meta-analytic evidence by Lim C. L. et al. (58), including 11 studies with 864 patients, confirmed these findings. Across all patient groups, prophylactic antibiotics did not significantly reduce the incidence of infected pancreatic necrosis, providing no conclusive support for their preventive use (58).

Additional analyses have explored broader clinical outcomes and specific antibiotic classes. Poropat et al. (59), analyzing 21 studies with 1,383 patients, suggested some benefits of prophylactic antibiotics, including reduced hospital stay, lower overall infection rates, and decreased extrapancreatic infections. In contrast, Guo et al. (60), evaluating prophylactic carbapenems in 3,864 patients across 7 studies, found significant reductions in overall infections (OR 0.27, *p* = 0.03) and complications (OR 0.48, *p* = 0.009), but no effect on infected pancreatic necrosis or mortality (OR 0.74, *p* = 0.24; OR 0.69, *p* = 0.17). These findings indicate that while prophylactic antibiotics may reduce certain systemic infections, they do not reliably prevent the most clinically relevant outcomes (60).

Overall, heterogeneity among studies—including differences in patient severity, antibiotic regimens, timing, and study endpoints—limits the ability to draw firm conclusions. Consequently, most clinical guidelines, including the American College of Gastroenterology (61), recommend antibiotics only when infection is suspected or confirmed and advise discontinuation if cultures or other diagnostics are negative. This reflects the ongoing uncertainty regarding routine prophylactic antibiotic use and highlights the need for well-designed, multicenter RCTs to define their optimal role in necrotizing pancreatitis.

### 6.4 Adverse effects of antibiotic prophylaxis

While antibiotic prophylaxis in necrotizing pancreatitis has been extensively investigated, its clinical utility remains controversial. Although some studies have suggested potential benefits in reducing certain infectious complications, the evidence is inconsistent, and routine prophylactic use is not supported by current guidelines. A key concern relates to potential adverse effects, including the emergence of multidrug-resistant organisms and the risk of fungal infections. Recent meta-analyses, however, did not demonstrate a statistically significant increase in fungal infections among patients receiving prophylactic antibiotics. For example, Ding N. et al. (57) reported fungal infections in 12.2% of patients in the antibiotic group versus 17.7% in controls (OR 0.95; 95% CI 0.30–3.03) (72), while Poropat G. et al. (59) observed fungal infections in 16 of 260 patients receiving prophylactic antibiotics versus 20 of 274 controls (RR 0.84; 95% CI 0.43–1.61). These findings suggest that although adverse events are possible, they may not be as frequent or severe as initially feared. Nevertheless, heterogeneity in patient populations, antibiotic regimens, timing, and study designs limits the reliability of these conclusions.

Beyond fungal infections, other potential adverse effects of prophylactic antibiotics include the development of antibiotic-resistant microorganisms and disruption of the gut microbiota. Widespread and prolonged use of antibiotics, including prophylactic administration, may contribute to the development of antibiotic-resistant microorganisms. Although resistant infections are relatively uncommon, their occurrence is associated with increased hospital stay and higher mortality, particularly in patients with pancreatic necrosis (62). Antibiotic therapy can also alter the composition and diversity of the gut microbiome, promoting colonization by opportunistic pathogens, exacerbating intestinal inflammation, and impairing immune responses. Such dysbiosis may delay the restoration of gut homeostasis after treatment and contribute to systemic infections and other complications (63, 64).

Given these considerations, current clinical guidelines do not recommend routine prophylactic use of systemic antibiotics in necrotizing pancreatitis. Evidence indicates that prophylaxis may reduce the incidence of certain infectious complications, but the magnitude of this effect is insufficient to justify its inclusion in standard practice (57, 59, 60). Further well-designed, multicenter studies are needed to clarify the balance between potential benefits and risks, particularly regarding fungal infections, antimicrobial resistance, and gut microbiota disruption. Such studies should standardize patient selection, antibiotic regimens, timing of administration, and study endpoints to reduce heterogeneity and improve generalizability. Moreover, investigating specific patient subgroups, such as those with extensive pancreatic necrosis or immunocompromised status, may identify populations that could selectively benefit from prophylactic antibiotics. Ultimately, a precision-medicine approach guided by biomarkers of infection risk and early clinical indicators may optimize antibiotic use, minimize adverse events, and improve outcomes in patients with necrotizing pancreatitis.

### 6.5 Selective digestive tract decontamination

Selective digestive tract decontamination (SDD) is a preventive strategy aimed at reducing intestinal colonization by potentially pathogenic microorganisms while preserving the normal anaerobic flora, which plays a key role in the host’s resistance to infection. The method was first proposed in 1979 for burn patients, after animal studies demonstrated that local antiseptic treatment alone was insufficient to prevent colonization by pathogenic bacteria (65). In 1980, SDD was clinically applied in granulocytopenic patients, where the use of a combination of topical and short-term systemic antibiotics significantly reduced the incidence of infections and mortality by suppressing aerobic gram-negative bacteria and yeasts while maintaining normal gut flora (66).

In the context of acute necrotizing pancreatitis, this approach is particularly relevant because bacterial infection of necrotic tissue occurs early and predominantly originates from intestinal translocation. Studies by H. G. Beger et al. (67) demonstrated that infected pancreatic necrosis is associated with high mortality, reaching up to 37.8%. These findings provided the rationale for considering SDD as a potential strategy to prevent infectious complications in patients with severe acute pancreatitis.

Current evidence suggests that SDD in patients with severe acute pancreatitis can reduce the incidence of infected pancreatic necrosis and overall mortality. The method is considered part of a comprehensive infection prevention strategy during the early stages of the disease, particularly when combined with additional measures such as strict infection control, patient monitoring, and rational antibiotic therapy (68–72). It is important to note potential limitations of SDD, including the risk of developing antibiotic-resistant flora and the need for careful antibiotic selection.

Given the diversity of approaches to infection prevention, a comparative overview is helpful to assess their relative value. A summary of the preventive strategies for infectious complications in acute necrotizing pancreatitis, with corresponding evidence grading and guideline recommendations, is presented in Table 1.

**Table 1:** Preventive strategies for infectious complications in acute necrotizing pancreatitis.

Preventive strategy	Strength of evidence (GRADE)	Guideline recommendation	Key notes
Early enteral nutrition	High	Recommended	Preserves gut barrier; reduces infectious complications
Preventive antibiotic therapy	Low-Moderate	Not routinely recommended	Decrease extrapancreatic infections, reduced hospital stays. No clear mortality benefit; may increase resistance
Probiotics	Low	Not recommended	Potential results inconsistent
Selective Digestive Tract Decontamination (SDD)	Moderate	Conditionally recommended	Reduces infected necrosis; early-stage use; risk of resistance

## 7 Conclusion

Future research should move beyond general recommendations toward precision approaches. Multicenter randomized controlled trials remain crucial to establish standardized protocols, but equal emphasis should be placed on risk stratification tools. Integration of inflammatory and immunological biomarkers (e.g., IL-6, procalcitonin, absolute lymphocyte count) with clinical scoring systems may improve the early identification of patients at highest risk of infected necrosis. Furthermore, advances in machine learning and predictive modeling could enable the development of dynamic decision-support tools that combine biomarker data, imaging findings, and clinical variables. Such personalized strategies would allow for the targeted use of preventive interventions—such as selective digestive decontamination or immunomodulatory nutrition—while avoiding unnecessary exposure in low-risk individuals. Ultimately, this paradigm shift from one-size-fits-all to stratified prevention may reduce complications, optimize resource allocation, and improve survival in necrotizing pancreatitis.
